# Supplementary material for: Characterization and Study on Fragmentation Pathways of a Novel Nerve Agent, ‘Novichok (A234)’, in Aqueous Solution by Liquid Chromatography–Tandem Mass Spectrometry
Source: Molecules. 2021 Feb 18;26(4):1059. doi: 10.3390/molecules26041059 (PMC7923011; doi:10.3390/molecules26041059)
Supplement: Supplementary file 1 [file molecules-26-01059-s001.pdf]

chemistry

MDPI

Supplementary Materials

Characterization and Study on Fragmentation pathways of a Novel Nerve Agent, 'Novichok (A234)', in Aqueous Solution by Liquid Chromatography-Tandem Mass Spectrometry

Jin Young Lee [1,*], Kyoung Chan Lim [1] and Hyun Suk Kim [1]

Affiliation Agency for Defense Development (ADD), P.O. Box 35-5, Yuseong-gu, Daejeon 305-600, Korea; marlintime@add.re.kr

* Correspondence: marlintime@add.re.kr; Tel.: +82-42-821-0483, Fax: +82-42-823-3400

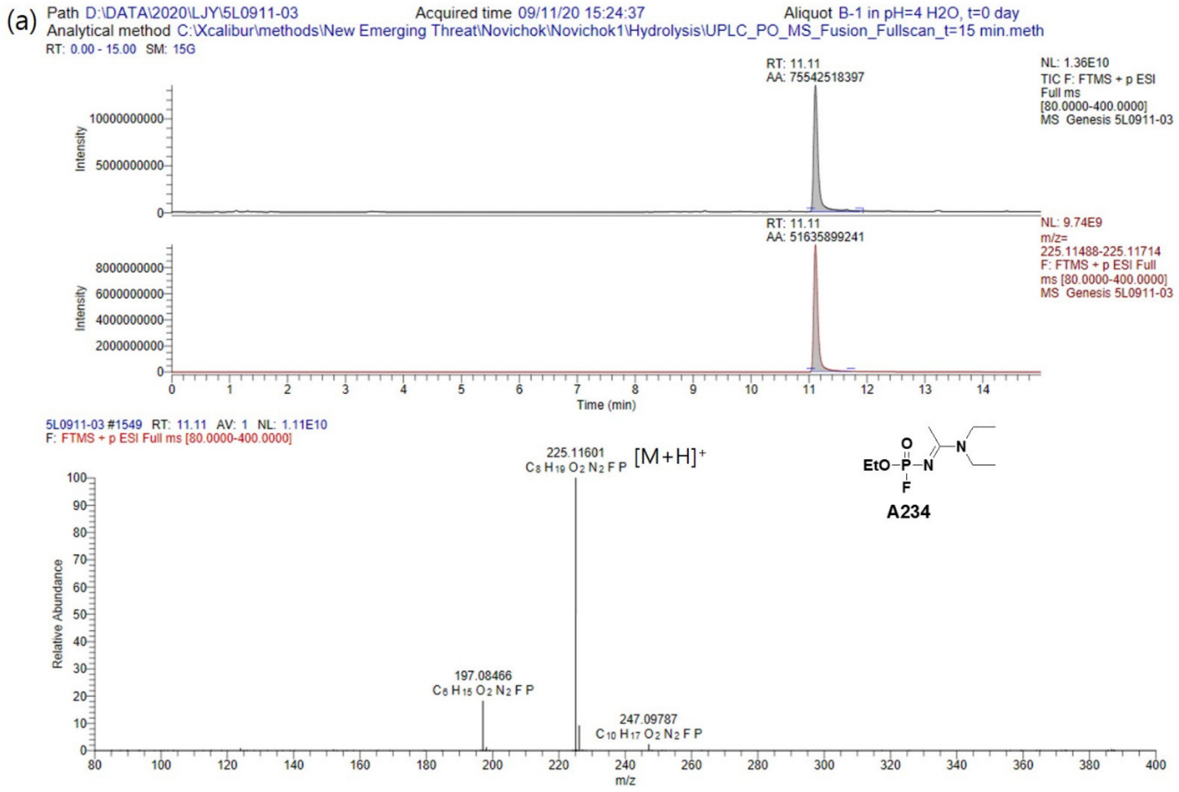

(b)

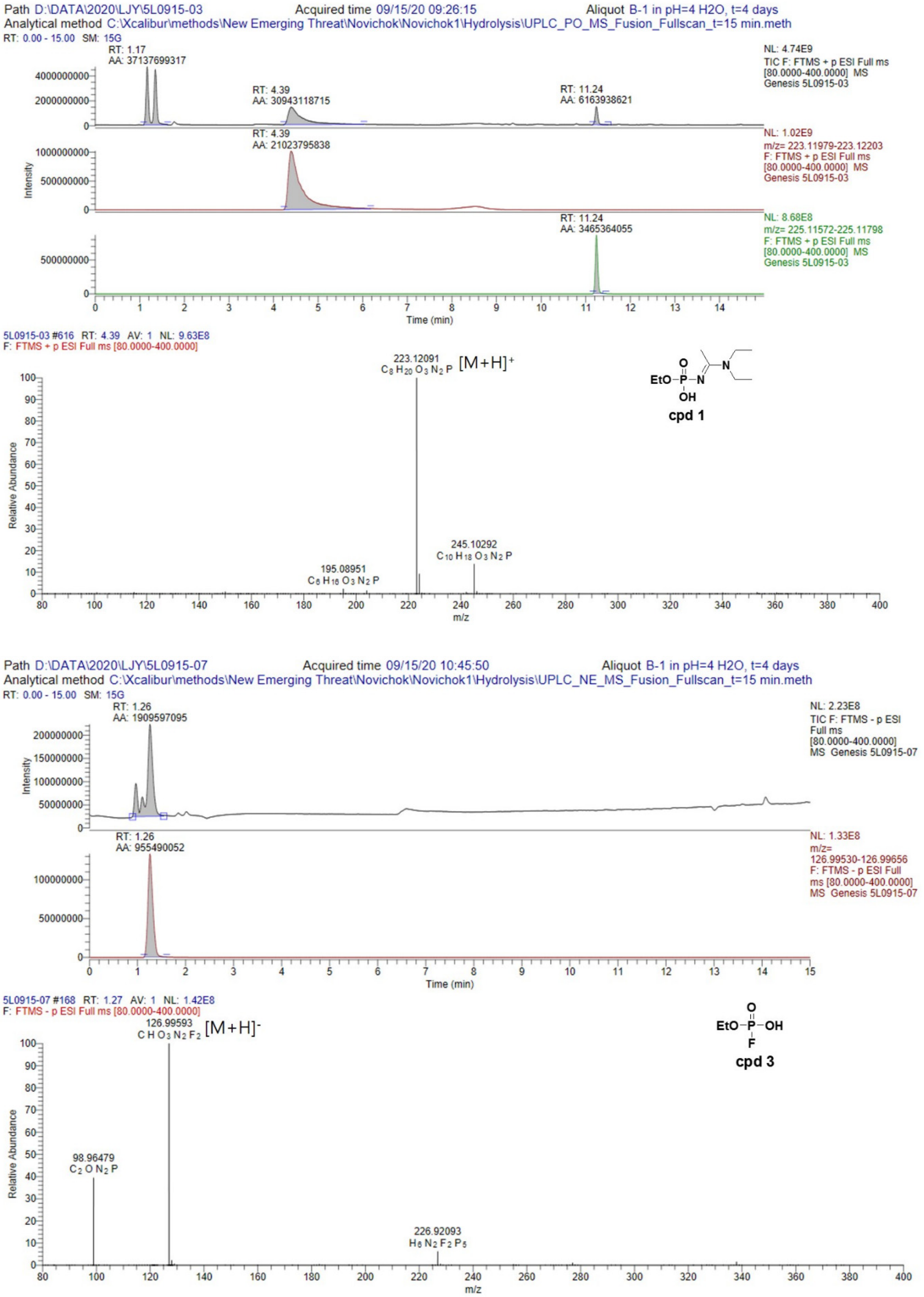
Path D:\DATA\2020\LJY\5L0915-03 Acquired time 09/15/20 09:26:15 Aliquot B-1 in pH=4 H2O, t=4 days
Analytical method C:\Xcalibur\methods\New Emerging Threat\Novichok\Novichok1\Hydrolysis\UPLC_PO_MS_Fusion_Fullscan_t=15 min.meth
RT: 0.00 - 15.00 SM: 15G
RT: 1.17
AA: 37137699317
RT: 4.39
AA: 30943118715
RT: 11.24
AA: 6163938621
NL: 4.74E9
TIC F: FTMS + p ESI Full ms [80.0000-400.0000] MS Genesis 5L0915-03
RT: 4.39
AA: 21023795838
NL: 1.02E9
m/z= 223.11979-223.12203
F: FTMS + p ESI Full ms [80.0000-400.0000] MS Genesis 5L0915-03
RT: 11.24
AA: 3465364055
NL: 8.68E8
m/z= 225.11572-225.11798
F: FTMS + p ESI Full ms [80.0000-400.0000] MS Genesis 5L0915-03
Intensity
Time (min)
5L0915-03 #616 RT: 4.39 AV: 1 NL: 9.63E8
F: FTMS + p ESI Full ms [80.0000-400.0000]
223.12091
C8 H20 O3 N2 P [M+H]+
195.08951
C6 H16 O3 N2 P
245.10292
C10 H18 O3 N2 P
Relative Abundance
m/z
EtO P N N OH
cpd 1
Path D:\DATA\2020\LJY\5L0915-07 Acquired time 09/15/20 10:45:50 Aliquot B-1 in pH=4 H2O, t=4 days
Analytical method C:\Xcalibur\methods\New Emerging Threat\Novichok\Novichok1\Hydrolysis\UPLC_NE_MS_Fusion_Fullscan_t=15 min.meth
RT: 0.00 - 15.00 SM: 15G
RT: 1.26
AA: 1909597095
NL: 2.23E8
TIC F: FTMS - p ESI Full ms [80.0000-400.0000] MS Genesis 5L0915-07
RT: 1.26
AA: 955490052
NL: 1.33E8
m/z= 126.99530-126.99656
F: FTMS - p ESI Full ms [80.0000-400.0000] MS Genesis 5L0915-07
Intensity
Time (min)
5L0915-07 #168 RT: 1.27 AV: 1 NL: 1.42E8
F: FTMS - p ESI Full ms [80.0000-400.0000]
126.99593
C H O3 N2 F2 [M+H]-
98.96479
C2 O N2 P
226.92093
H8 N2 F2 P5
Relative Abundance
m/z
EtO P OH F
cpd 3

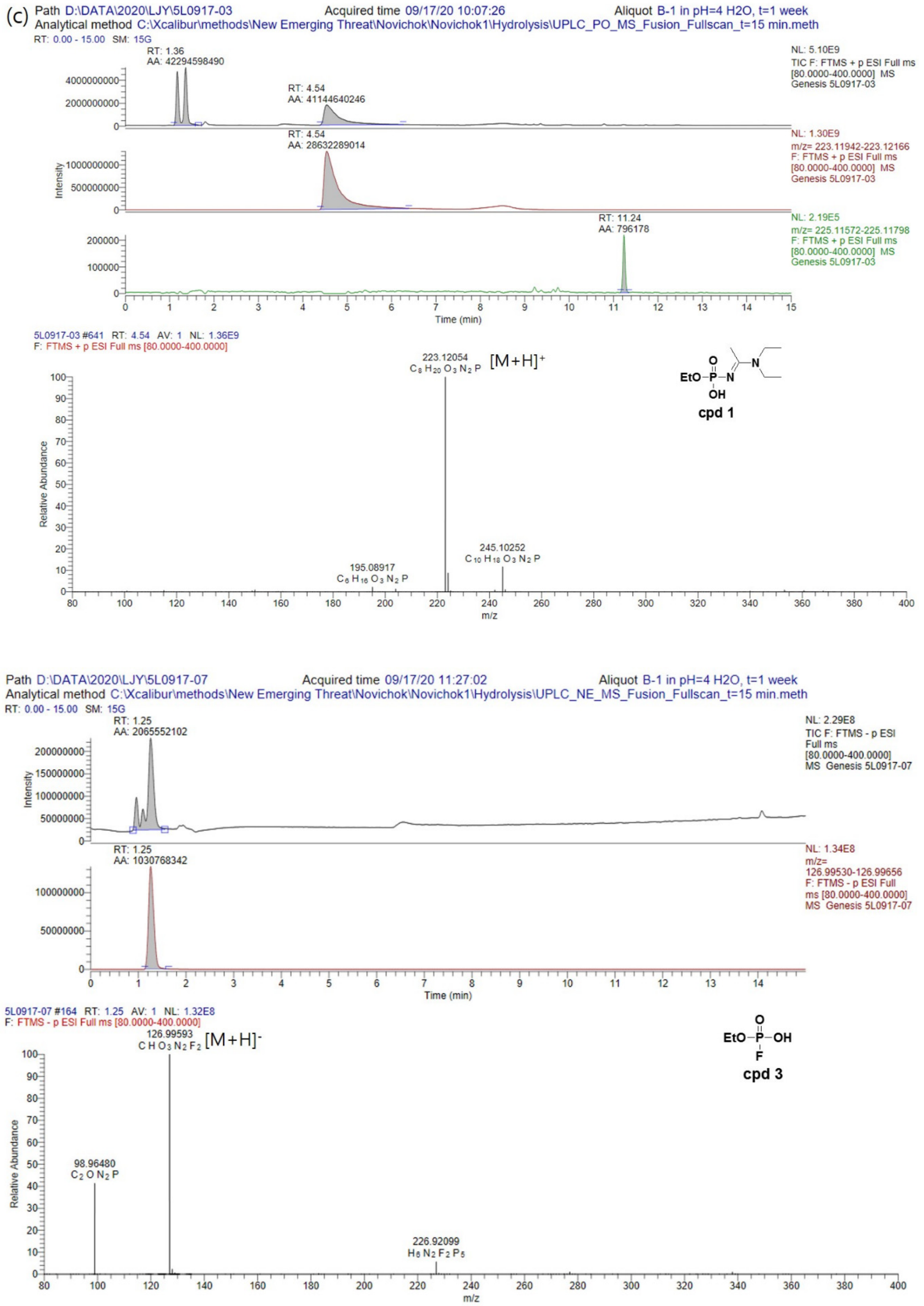

Supplemental figure 1. Time profile of A234 in an acidic aqueous solution for 1 week. (a) t = 0 h, (b) t = 4 days, (c) t = 1 week.

(a)

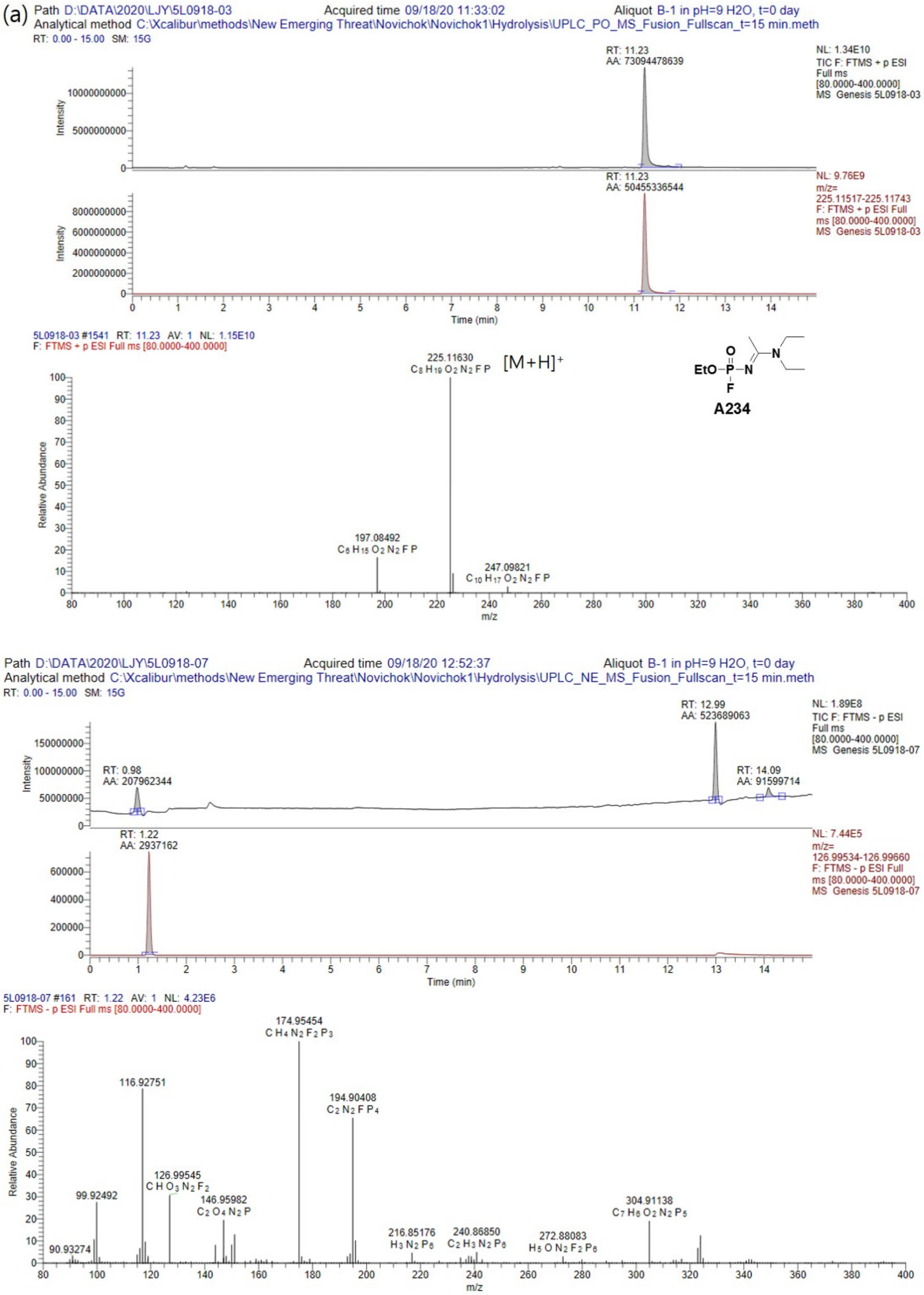
Path D:\DATA\2020\LJY\5L0918-03 Acquired time 09/18/20 11:33:02 Aliquot B-1 in pH=9 H2O, t=0 day
Analytical method C:\Xcalibur\methods\New Emerging Threat\Novichok\Novichok1\Hydrolysis\UPLC_PO_MS_Fusion_Fullscan_t=15 min.meth
RT: 0.00 - 15.00 SM: 15G
RT: 11.23
AA: 73094478639
NL: 1.34E10
TIC F: FTMS + p ESI Full ms [80.0000-400.0000] MS Genesis 5L0918-03
RT: 11.23
AA: 50455336544
NL: 9.76E9
m/z= 225.11517-225.11743 F: FTMS + p ESI Full ms [80.0000-400.0000] MS Genesis 5L0918-03
Intensity
Time (min)
5L0918-03 #1541 RT: 11.23 AV: 1 NL: 1.15E10
F: FTMS + p ESI Full ms [80.0000-400.0000]
225.11630
C8 H19 O2 N2 F P
[M+H]+
197.08492
C6 H15 O2 N2 F P
247.09821
C10 H17 O2 N2 F P
EtO
A234
Relative Abundance
m/z
Path D:\DATA\2020\LJY\5L0918-07 Acquired time 09/18/20 12:52:37 Aliquot B-1 in pH=9 H2O, t=0 day
Analytical method C:\Xcalibur\methods\New Emerging Threat\Novichok\Novichok1\Hydrolysis\UPLC_NE_MS_Fusion_Fullscan_t=15 min.meth
RT: 0.00 - 15.00 SM: 15G
RT: 12.99
AA: 523689063
NL: 1.89E8
TIC F: FTMS - p ESI Full ms [80.0000-400.0000] MS Genesis 5L0918-07
RT: 0.98
AA: 207962344
RT: 14.09
AA: 91599714
RT: 1.22
AA: 2937162
NL: 7.44E5
m/z= 126.99534-126.99660 F: FTMS - p ESI Full ms [80.0000-400.0000] MS Genesis 5L0918-07
Time (min)
5L0918-07 #161 RT: 1.22 AV: 1 NL: 4.23E6
F: FTMS - p ESI Full ms [80.0000-400.0000]
174.95454
C H4 N2 F2 P3
116.92751
194.90408
C2 N2 F P4
126.99545
C H O3 N2 F2
99.92492
146.95982
C2 O4 N2 P
304.91138
C7 H6 O2 N2 P5
216.85176
H3 N2 P6
240.86850
C2 H3 N2 P6
272.88083
H5 O N2 F2 P6
90.93274
Relative Abundance
m/z

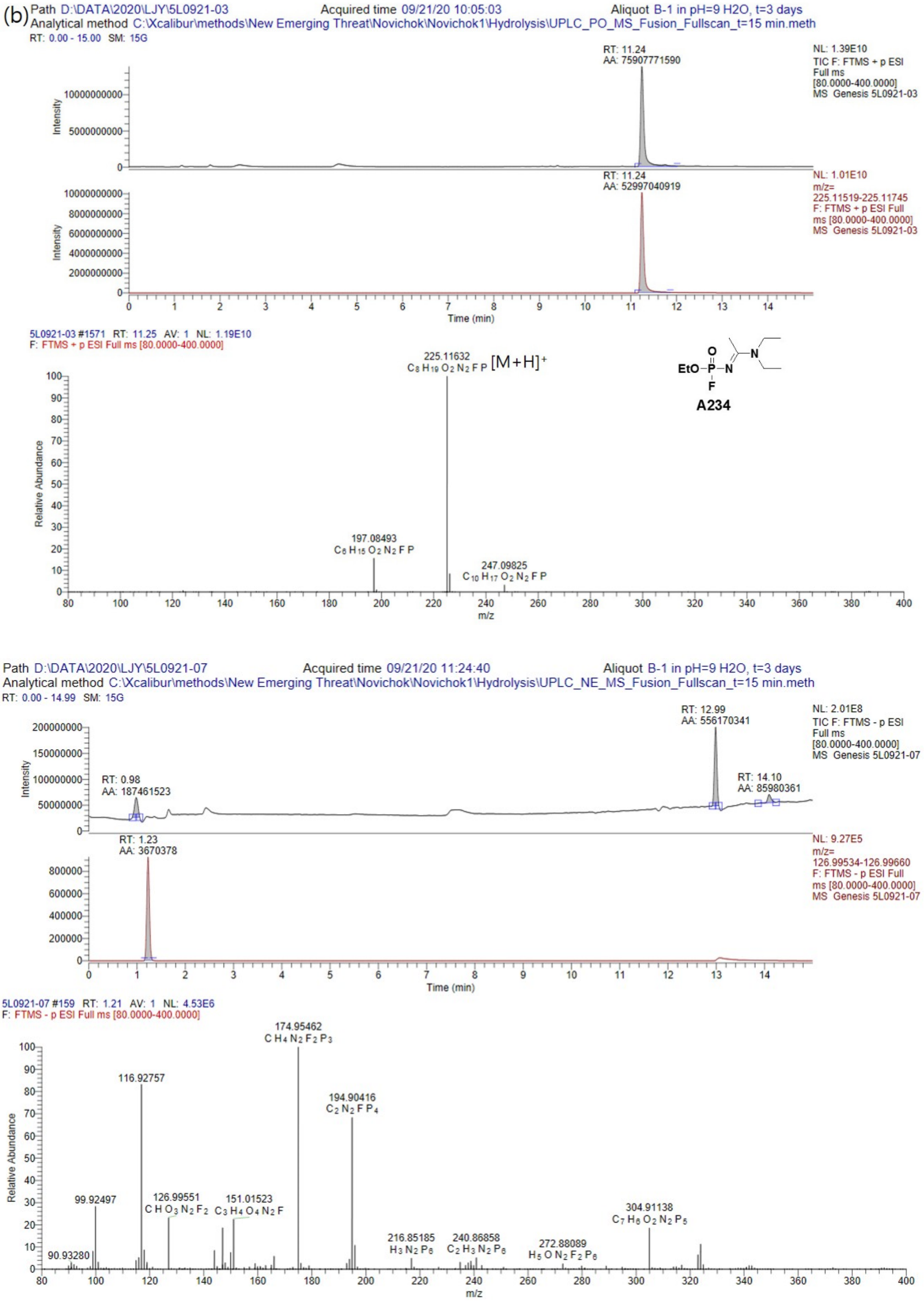
(b)
Path D:\DATA\2020\LJY\5L0921-03 Acquired time 09/21/20 10:05:03 Aliquot B-1 in pH=9 H2O, t=3 days
Analytical method C:\Xcalibur\methods\New Emerging Threat\Novichok\Novichok1\Hydrolysis\UPLC_PO_MS_Fusion_Fullscan_t=15 min.meth
RT: 0.00 - 15.00 SM: 15G
RT: 11.24 AA: 75907771590
NL: 1.39E10 TIC F: FTMS + p ESI Full ms [80.0000-400.0000] MS Genesis 5L0921-03
RT: 11.24 AA: 52997040919
NL: 1.01E10 m/z= 225.11519-225.11745 F: FTMS + p ESI Full ms [80.0000-400.0000] MS Genesis 5L0921-03
Intensity
Time (min)
5L0921-03 #1571 RT: 11.25 AV: 1 NL: 1.19E10
F: FTMS + p ESI Full ms [80.0000-400.0000]
225.11632 C8 H19 O2 N2 F P [M+H]+
197.08493 C6 H15 O2 N2 F P
247.09825 C10 H17 O2 N2 F P
EtO P N N O F
A234
Relative Abundance
m/z
Path D:\DATA\2020\LJY\5L0921-07 Acquired time 09/21/20 11:24:40 Aliquot B-1 in pH=9 H2O, t=3 days
Analytical method C:\Xcalibur\methods\New Emerging Threat\Novichok\Novichok1\Hydrolysis\UPLC_NE_MS_Fusion_Fullscan_t=15 min.meth
RT: 0.00 - 14.99 SM: 15G
RT: 12.99 AA: 556170341
NL: 2.01E8 TIC F: FTMS - p ESI Full ms [80.0000-400.0000] MS Genesis 5L0921-07
RT: 0.98 AA: 187461523
RT: 14.10 AA: 85980361
RT: 1.23 AA: 3670378
NL: 9.27E5 m/z= 126.99534-126.99660 F: FTMS - p ESI Full ms [80.0000-400.0000] MS Genesis 5L0921-07
Intensity
Time (min)
5L0921-07 #159 RT: 1.21 AV: 1 NL: 4.53E6
F: FTMS - p ESI Full ms [80.0000-400.0000]
174.95462 C H4 N2 F2 P3
116.92757
194.90416 C2 N2 F P4
99.92497
126.99551 C H O3 N2 F2
151.01523 C3 H4 O4 N2 F
90.93280
216.85185 H3 N2 P6
240.86858 C2 H3 N2 P6
272.88089 H5 O N2 F2 P6
304.91138 C7 H6 O2 N2 P5
Relative Abundance
m/z

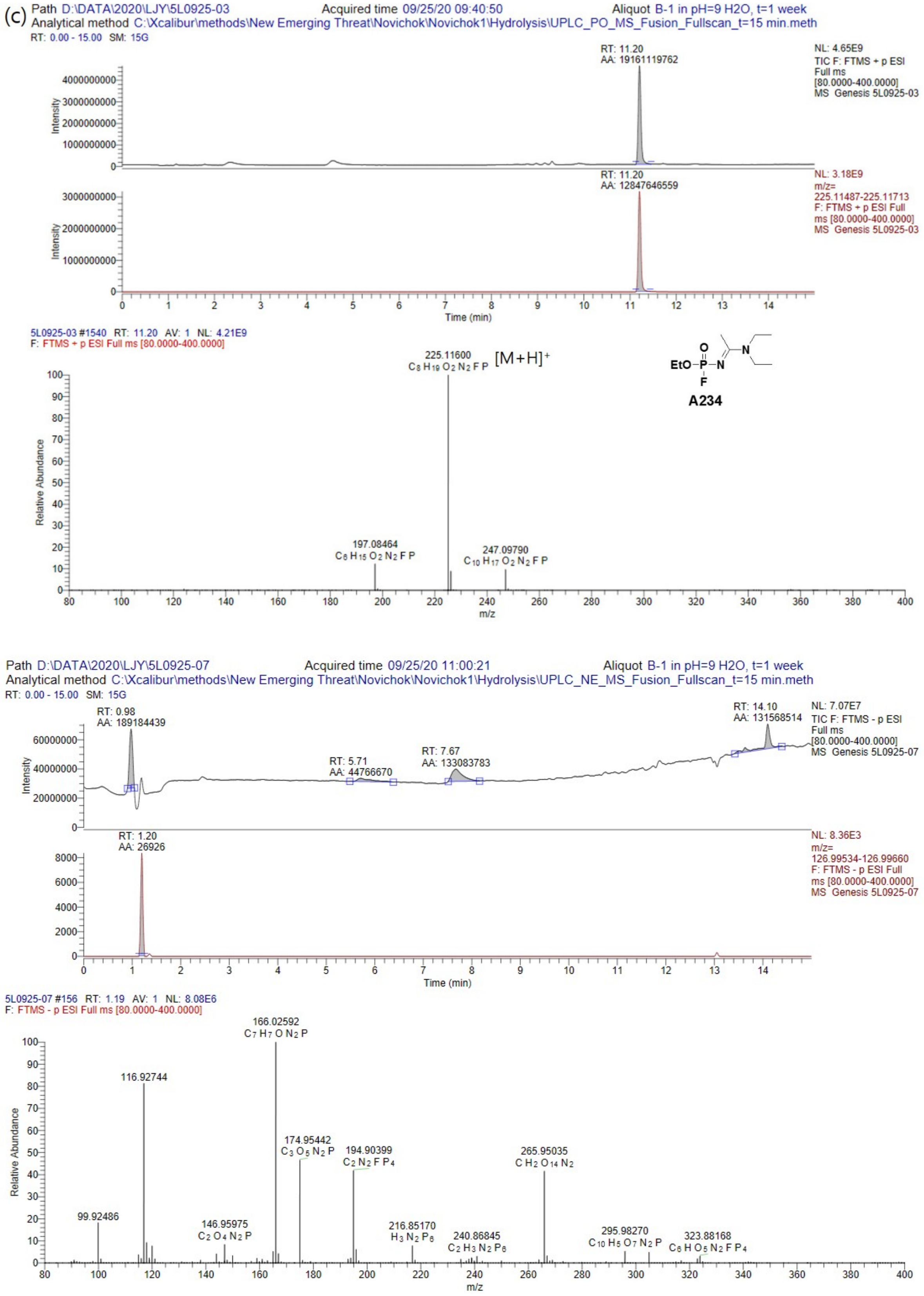
(c)
Path D:\DATA\2020\LJY\5L0925-03 Acquired time 09/25/20 09:40:50 Aliquot B-1 in pH=9 H2O, t=1 week
Analytical method C:\Xcalibur\methods\New Emerging Threat\Novichok\Novichok1\Hydrolysis\UPLC_PO_MS_Fusion_Fullscan_t=15 min.meth
RT: 0.00 - 15.00 SM: 15G
RT: 11.20
AA: 19161119762
NL: 4.65E9
TIC F: FTMS + p ESI Full ms [80.0000-400.0000]
MS Genesis 5L0925-03
RT: 11.20
AA: 12847646559
NL: 3.18E9
m/z= 225.11487-225.11713
F: FTMS + p ESI Full ms [80.0000-400.0000]
MS Genesis 5L0925-03
Intensity
Time (min)
5L0925-03 #1540 RT: 11.20 AV: 1 NL: 4.21E9
F: FTMS + p ESI Full ms [80.0000-400.0000]
225.11600
C8 H19 O2 N2 F P
[M+H]+
197.08464
C6 H15 O2 N2 F P
247.09790
C10 H17 O2 N2 F P
EtO
A234
Relative Abundance
m/z
Path D:\DATA\2020\LJY\5L0925-07 Acquired time 09/25/20 11:00:21 Aliquot B-1 in pH=9 H2O, t=1 week
Analytical method C:\Xcalibur\methods\New Emerging Threat\Novichok\Novichok1\Hydrolysis\UPLC_NE_MS_Fusion_Fullscan_t=15 min.meth
RT: 0.00 - 15.00 SM: 15G
RT: 0.98
AA: 189184439
RT: 5.71
AA: 44766670
RT: 7.67
AA: 133083783
RT: 14.10
AA: 131568514
NL: 7.07E7
TIC F: FTMS - p ESI Full ms [80.0000-400.0000]
MS Genesis 5L0925-07
RT: 1.20
AA: 26926
NL: 8.36E3
m/z= 126.99534-126.99660
F: FTMS - p ESI Full ms [80.0000-400.0000]
MS Genesis 5L0925-07
Intensity
Time (min)
5L0925-07 #156 RT: 1.19 AV: 1 NL: 8.08E6
F: FTMS - p ESI Full ms [80.0000-400.0000]
166.02592
C7 H7 O N2 P
116.92744
174.95442
C3 O5 N2 P
194.90399
C2 N2 F P4
265.95035
C H2 O14 N2
99.92486
146.95975
C2 O4 N2 P
216.85170
H3 N2 P6
240.86845
C2 H3 N2 P6
295.98270
C10 H5 O7 N2 P
323.88168
C6 H O5 N2 F P4
Relative Abundance
m/z

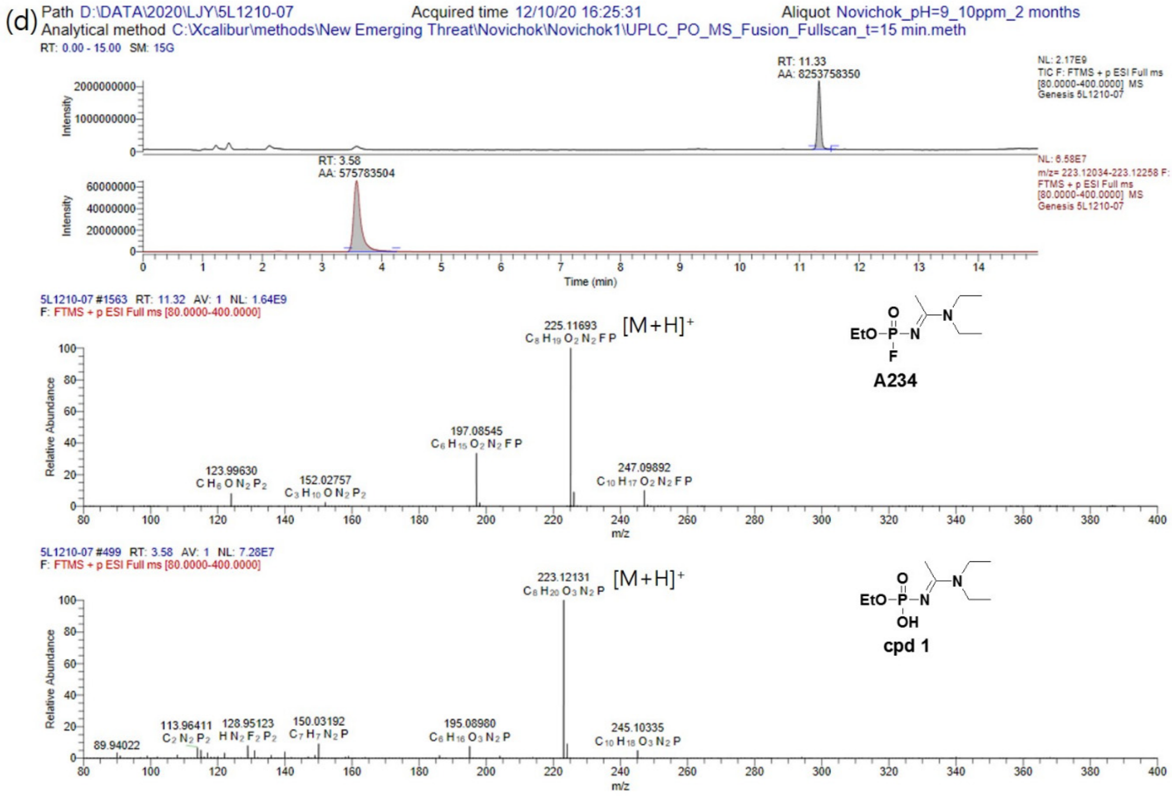

Supplemental figure 2. Time profile of A234 in a basic aqueous solution for 1 week. (a) t = 0 h, (b) t = 3 days, (c) t = 1 week, (d) t = 2 months.
